# Analysis of the Relationship between Elite Wrestlers’ Leg Strength and Balance Performance, and Injury History

**DOI:** 10.3390/sports6020035

**Published:** 2018-04-17

**Authors:** Sezen Çimen Polat, Celal Bulgay, İmdat Yarım, Halil İbrahim Cicioğlu, Ebru Çetin

**Affiliations:** Faculty of Sports Sciences, Gazi University, Ankara 06330, Turkey; celalbulgay@hotmail.com (C.B.); imdat@gazi.edu.tr (İ.Y.); cicioglu@gazi.edu.tr (H.İ.C.); cetinebruu@gmail.com (E.Ç.)

**Keywords:** free style wrestling, disability, strength, balance

## Abstract

The purpose of this study is to examine the correlation between leg power and balance performance in elite wrestlers and injury history. In the research group, there are 18 elite freestyle male wrestlers at the ages of 24.27 ± 3.18 years, with a height of 171.86 ± 5.44 cm and a body weight of 79.27 ± 11.16 kg. Information on the injury history of the athletes’ upper legs for the past year was collected via interviews with the club’s physiotherapist. Laboratory tests to measure performance assessed height, body weight, Y balance and isokinetic leg strength. Data obtained from the study are presented as mean and standard deviation. The test of normality was carried out by the Shapiro-Wilk test. The Pearson Correlation Test was performed for all parameters with normal distribution, and significance level was accepted as *p* < 0.05. It was found that there is a relationship between the wrestlers’ right leg ratio and hamstring strength and injury history. However, there is no statistically significant relationship between left leg hamstring, quadriceps, ratio, right leg quadriceps, or right and left leg balance performance, and injury history. The resulting data shows that the proportioning between hamstring and quadriceps muscles in freestyle wrestlers’ upper leg strength values is not ideal. This finding provides evidence that injury risk increases with the additional impact of loss of strength.

## 1. Introduction

Injury risk is unavoidable in sports and exercise, as are their long-term health benefits; thus, sports injury is a condition that requires precaution [1]. Risk factors in sports can be examined in two groups: contact injuries and non-contact injuries. Contact injury occurs when an athlete contacts with another athlete or object during competitions and training. Non-contact injuries are endogenous injuries like neuromuscular disorders. Such injuries are the focus of prevention initiatives, and managing identified risk factors plays an important role [2]. A study on the probability of injury found that the sports with most frequent injuries are football with 10%, wrestling with 6%, handball and boxing with 3%, athletics with 1% and skiing with 0.5% probability [3]. Another study on the sports with the most frequent injuries found that, of 6030 pre-season injuries in the English professional football league, 23% were related to thighs and 17% were related to knees [4]. It can be seen that wrestlers rank second in terms of injury risk, because wrestling is among the sports that require enormous effort and have a high risk of injury. Wrestling is a combat sport in which two opponents struggle hand-to-hand in order to pin or press each other’s shoulders to the mat or ground, or gain technical advantage with all of their physiological and psychological power, without using any tools or other means, in a limited area for a given period in accordance with certain rules [5]. In a wrestling competition, wrestlers use all parts of their body to defeat the opponent. Meanwhile, the biomechanical forces applied to the athletes can cause damage in different parts of the body [6]. Sprains, strains and bruises are reported as the most common injuries seen among wrestlers [7]. In their study on 319 active athletes in various sports, Uğur et al. found that injury risk decreases as the strength of leg muscle increases [8]. In addition, hamstring/quadriceps ratio (H/Q) is used as a determinant in the prevention of injury, as well as being a muscular balance indicator. Imbalance between agonist and antagonist muscle groups increases the risk of injury, and particularly the weakness of the hamstring muscle group is known to provide a basis for injuries [9]. Studies conducted to prevent injuries suggest that the strength-balance relationship, particularly, can be influential [10]. It is stated that balance exercises are quite effective in terms of neuromuscular and functional performance. It is also known that they are critically important in the rehabilitation process for going back to training and competitions after sports injuries [11]. Studies conducted on different branches maintain that eccentric quadriceps strength and low eccentric flexor/extensor ratio are related to an increase in anterior cruciate ligament injury risk, and H/Q values should be determined to prevent injuries, and for rehabilitation [12,13]. Research indicates that there is a high risk of injury in wrestling. Consequently, it can be said that these studies have mostly focused on the classification of injuries and disablement types. However, the relationships between injury types in different sports and different variables can also be examined. The relationship between strength, balance and sports injuries has a significant role in the prevention of injuries and their treatment in a short time in wrestling. Therefore, to assess the relationship between strength and balance parameters and improvement rates in both parameters in wrestlers, this study examines the relationship between elite wrestlers’ leg strength, balance performance, and injury history.

## 2. Material and Methods

### 2.1. Participants

The study was conducted on 18 elite freestyle male wrestlers aged 24.27 ± 3.18 years, with heights of 171.86 ± 5.44 cm and body weights of 79.27 ± 11.16 kg. The wrestlers are from the same freestyle wrestling team in Ankara, and have been engaged in wrestling for 9.12 ± 1.24 years. In the study, their height, body weight, body mass index, body fat percent, Y balance test and isokinetic leg strength were assessed (Table 1). After that, information on the injury history of the athletes’ upper legs for the previous 6 months was collected via interviews with the club’s physiotherapist. While 7 wrestlers said they had never sustained an injury before, the 11 wrestlers with an injury history reported that 3 had meniscus injury, 4 of them had a herniated disc, and the remaining 4 had muscle rupture, and they were all treated. The test procedures were explained to the wrestlers in detail before application. Each wrestler signed a consent form to allow the use of these measurements and injury history in an academic study, and participation in the research was on a voluntary basis.

### 2.2. Procedures

Height and body weight were assessed using stadiometer (Seca) height and body weight scale without any footwear. Isokinetic leg strength was assessed using ‘Isomed 2000, Germany’ equipment. The subjects’ isokinetic right and left leg quadriceps, hamstring and quadriceps-hamstring strength ratios were measured at an angular speed of 60 degrees/second, with 5 repetitions. For each assessment, the subjects underwent 5 weightless trials before the test. The participants were encouraged with verbal expressions so that they would deliver a better performance during isokinetic strength measurement. Balance performance was measured through a Y balance test. The participant is at the center of the test, standing on one leg in balance while reaching out with the other leg to the farthest points of lines prepared in 3 directions—anterior, posteromedial and posterolateral [2,3,4,5,6,7,8,9,10,11,12,13,14]. The best performance reached at the end of 3 assessments is recorded. Information on injuries was obtained from the documents received from the club’s physiotherapist.

### 2.3. Statistical Analysis

Data obtained in the study are presented as average and standard deviation. The Shapiro-Wilk test was performed to assess normality. For all parameters displaying normal distribution, the Pearson Correlation test was performed. The level of significance was accepted as *p* < 0.05.

## 3. Results

The study examined the relationship between leg strength and balance performance in elite wrestlers and injury history. At the end of this examination, it was found that there is a relationship between wrestlers’ right leg ratio and hamstring strength and injury history. However, no statistically significant relationship is found between left leg hamstring, quadriceps ratio, right leg quadriceps and right/left leg balance performance, and injury history.

## 4. Discussion and Conclusions

This study examined the relationship between leg strength and balance performance and injury history in elite wrestlers. It was found that there is a relationship between wrestlers’ right leg ratio and hamstring strength and injury history. However, no statistically significant relationship is found between left leg hamstring, quadriceps ratio, right leg quadriceps and right/left leg balance performance, and injury history (Table 2, Table 3, Table 4 and Table 5). In a literature review, we found many studies on the isokinetic moment ratios of hamstring and quadriceps muscle groups and their impact on muscle imbalances. These studies underline the importance of the fact that disproportionate use of strength between agonist and antagonist muscles increases the risk of injury. H/Q ratio is used to examine the similarities of moment-speed patterns between hamstring and quadriceps, and to assess the functional competence and muscle balance of the knee. H/Q ratio depends on speed and location, and reflects the tendency to injury [13]. Beneka et al. state that the ideal balance between quadriceps and hamstring muscle strengths is 3/2. Pathological boundaries for the Q angle are not certain [15]. Some researchers maintain that Q angles higher than 20 degrees may cause anterior knee pain [16]. Şenışık et al. conducted a study on the impact of isokinetic muscle strength on anterior cruciate ligament injury risk in professional football players. Isokinetic strength in the players’ knee extensor and flexor muscles was assessed using Cybex Norm dynamometer at speeds of 60 and 300°/s. During the 30-month recording of injuries, 11 non-contact anterior cruciate ligament injuries were observed. While injured players had higher eccentric quadriceps strength at 60°/s. compared to the uninjured, no statistically significant difference was found between the groups in terms of other strength values. According to the results of the study, a relationship was found between eccentric quadriceps strength and low eccentric flexor/extensor ratio, and increasing risk of anterior cruciate ligament injuries [12]. Malliaropoulos et al. recommended strengthening hamstring muscles for the prevention of sports injuries. In his study, which associated quadriceps and hamstring muscle strength in wrestlers and football players with the tendency to injury [17], Tortop measured the subjects’ knee flexion/extension muscle strength at angular speeds of 60°/s. and 180°/s. The study found a decline in H/Q ratios as the league and quality improved for the athletes. It was also found that H/Q ratio increases in direct proportion to angular speed. The values obtained in the study were not enough to cause first-degree injuries in the athletes and were within normally accepted limits. In other words, it was found that there was no disproportionate strength development or serious injury tendency in the athletes [18]. In their study on 28 elite football players, Dauty et al. state that 15 players were injured in the hamstring muscle group in 2 years, and the test was performed after their recovery. They found that unilateral concentric quadriceps-hamstring ratios and eccentric hamstring, concentric quadriceps ratios were less than 0.6, and the difference between right and left legs was less than 10%. In their study examining the relationship between muscle strength and Q angle in different sports branches [19], Atamaz et al. measured hamstring and quadriceps muscle strength in swimmers and football players, and found no significant difference between the groups for any parameters except hamstring muscle strength at an angular speed of 60°/s [16]. This study indicated that Q angle may not be affected by sports activity in different sports. All of these studies examine the relationship between strength and injury. As can be seen from this study, there is a relationship between strength and injury risk. However, there is not much evidence of a relationship between strength, balance and injury. The relationship with balance performance might be decisive for both strength and injury [10]. Therefore, this study also examines the relationship between balance performance and injury. According to the results of the assessment, no statistically significant relationship is found between right and left leg balance performance and injury history. There are some studies that examine only injury and balance (Table 4 and Table 5). Ulusoy examined the relationship between post-rehabilitation isokinetic knee muscle strength and dynamic balance, and found no relationship between Y test posterolateral reach distance and muscle strength in healthy individuals. It is indicated that there is a positive relationship between different directions of dynamic balance and isokinetic strengths in different muscle groups in individuals who have had an anterior cruciate ligament operation using a hamstring autograft [20]. Moreover, in studies examining the relationship between balance and isokinetic muscle strength [21,22], no significant relationship has been found between hamstring and quadriceps muscle strength and static balance in individuals who have an anterior cruciate ligament injury. Another study by Gonell et al. attempted to determine whether balance test is applicable to identifying subjects susceptible to soft tissue damage among professional football players. The anterior, posteromedial and posterolateral reach distances of 74 football players were recorded and physiotherapists documented how long they did not play football due to injury. Each extension distance, right/left reach distance difference and compound reach distance was assessed using probability rates and regression analysis. The results indicated that the possibility of self-injury almost doubled for players with scores lower than the average score in each reach direction [2]. Similarly, McLeod et al. and Koenig and Puckree revealed the relationship between poor balance competence and increasing risk of injury in athletes [23,24]. On the basis of the results of our assessment of leg strength and balance performance and injury history in elite wrestlers, a relationship is found between the wrestlers’ right leg ratio and hamstring strength, and injury history (Table 2 and Table 3). Data obtained in the study indicates that the ratio of hamstring and quadriceps muscles in freestyle wrestlers’ leg strength is far from ideal (Table 1). This finding can provide practitioners with evidence that injury risk can increase with the additional impact of the loss of strength and balance. Consequently, it is recommended that to avoid higher injury risk, wrestlers should pay attention to the relationship between strength and balance, and engage more in exercises that improve these parameters.

## Figures and Tables

**Table 1:** Descriptive statistics.

Parameters	Average	Standard Deviation (±)
Age (year)	24.27	3.18
Height (cm)	171.86	5.44
Body weight (kg)	79.27	11.16
Body mass index (kg/m^2^)	23.34	2.21
Body fat percent (%)	13.61	5.83
Right hamstring 60°·s^−1^ (N.m^−1^)	142.13	22.75
Right quadriceps 60°·s^−1^ (N.m^−1^)	223.27	35.95
Right ratio (%)	62.85	6.23
Left hamstring 60°·s^−1^ (N.m^−1^)	139.97	22.62
Left quadriceps 60°·s^−1^ (N.m^−1^)	234.77	49.24
Left ratio (%)	60.02	7.98
Right anterior balance (cm)	79.47	5.13
Right posteromedial balance (cm)	108.11	9.22
Right posterolateral balance (cm)	99.93	8.71
Left anterior balance (cm)	78.32	5.47
Left posteromedial balance (cm)	105.55	6.19
Left posterolateral balance (cm)	100.36	9.72

**Table 2:** Relationship between injury and right leg isokinetic strength.

*N* = 18	Right Hamstring		Right Quadriceps		Right Ratio	
Parameter	Correlation	P	Correlation	P	Correlation	P
Injury	0.703	0.005 *	−0.330	0.271	0.703	0.005 *

* *p* < 0.05.

**Table 3:** Relationship between injury and left leg isokinetic strength.

*N* = 18	Left Hamstring		Left Quadriceps		Left Ratio	
Parameter	Correlation	P	Correlation	P	Correlation	P
Injury	−0.082	0.789	−0.289	0.339	0.496	0.085

**Table 4:** Relationship between injury and right balance parameter.

*N* = 18	Right Anterior Balance		Right Posteromedial Balance		Right Posterolateral Balance	
Parameter	Correlation	P	Correlation	P	Correlation	P
Injury	0.374	0.208	0.186	0.543	0.269	0.375

**Table 5:** Relationship between injury and left balance parameter.

*N* = 18	Right Anterior Balance		Right Posteromedial Balance		Right Posterolateral Balance	
Parameter	Correlation	P	Correlation	P	Correlation	P
Injury	0.394	0.183	0.248	0.414	−0.021	0.947

## References

[1] Fuller C.W., Ward C.J. (2008). An Empirical Approach for Defining Acceptable Levels of Risk: A Case Study in Team Sports. Inj. Prev..

[2] Gonell A.C., Romero J.A.P., Soler L.M. (2015). Relationship between the Y Balance Test Scores and Soft Tissue Injury Incidence in a Soccer Team. Int. Sports Phys. Ther..

[3] Sakallı F.M.H. (2008). Sporda Sporcuların Yaralanması Ve Risk Faktörleri. Fırat Sağlık Hizmetleri Dergisi.

[4] Woods C., Hawkins R., Hulse M., Hodson A. (2002). The Football Association Medical Research Programme: An Audit of Injuries in Professional Football Analysis of Preseason Injuries. Br. J. Sports Med..

[5] Açak M. (2005). Beden Eğitimi Öğretmeninin El Kitabı.

[6] Kabak B., Karanfilci M., Karakuyu N.A. (2017). Comparison of Sports Injuries in Judo and Wrestling Sports. J. Sports Perform. Res..

[7] Miller L.E., Pierson L.M., Nickols-Richardson S.M., Wootten D.F., Selmon S.E., Ramp W.K., Herbert W.G. (2006). Knee Extensor And Flexor Torque Development With Concentric And Eccentric Isokinetic Training. Res. Q. Exerc. Sport.

[8] Uğur M., Can S., Şenel K. (1999). The Effect of THE Muscle Power and the Hand Preference on Injuries in Various Sport Branches. Atatürk Üniversitesi Beden Eğitimi ve Spor Bilimleri Dergisi.

[9] Evangelidis P.E., Massey G.J., Ferguson R.A., Wheeler P.C., Pain M.T., Folland J.P. (2016). The Functional Significance of Hamstrings Composition: Is It Really A “Fast” Muscle Group. Scand. J. Med. Sci. Sports.

[10] Alpay B. (2000). Türkiye’de Serbest Güreş a Milli Takımı İle Niğde Üniversitesi Güreş Takımı Güreşçilerinin Bazı Dolaşım Ve Solunum Parametrelerinin Karşılaştırılması.

[11] Ateş B., Çetin E., Yarım İ. (2017). Balance Ability and Balance Training in Female Athletes. Gaziantep Üniversitesi Spor Bilimleri Dergisi.

[12] Şenışık S., Özgürbüz C., Ergün M. (2010). Elit Futbolcularda İzokinetik Kas Kuvveti Ve Ön Çapraz Bağ Yaralanması Arasındaki İlişki. Turk. J. Sports Med..

[13] Yenigün Ö., Çolak T., Bamaç B., Yenigün N., Özbek A., Bayazıt Çolak E. (2008). The Determination of Isokinetic Performance Values of Knee Joint and Hamstring (Flexor)/Quadriceps (Extensor) Ratios Differences in Volleyball Players. J. Hum. Sci..

[14] Şahin G., Şeker H., Yeşilırmak M., Çadır A. (2015). Denge Diski Egzersizlerinin Dinamik Denge Ve Duruş Kontrolü Üzerindeki Etkisinin İncelenmesi. Spor Ve Performans Araştırmaları Dergisi.

[15] Benaka A., Malliou P., Gioffsidou A. (2003). Restoration of Muscles Imbalances with a Spesific Strength Training Programme in Young Soccer Players. Science and Football V, the Proceedings of the Fifth World Congress on Sports Science and Football, Lisbon, Portugal, 11‒15 April 2003.

[16] Atamaz F., Özdedeli S., Canüzmez A., Özçaldıran B., Durmaz B. Farklı Spor Dallarında Kas Kuvveti İle Q Açısı İlişkisi. http://www.academia.edu/7942241/FARKLI_SPOR_DALLARINDA_KAS_KUVVET%C4%B0_%C4%B0LE_Q_A%C3%87ISI_%C4%B0L%C4%B0%C5%9EK%C4%B0S%C4%B0.

[17] Malliaropoulos N., Mendiguchia J., Pehlivanidis H., Papadopoulou S., Valle X., Malliaras P., Maffulli N. (2012). Hamstring Exercises For Track and Field Athletes: Injury and Exercise Biomechanics, and Possible Implications For Exercise Selection and Primary Prevention. Br. J. Sports Med..

[18] Tortop Y. (2009). Güreşci ve Futbolcuların Quadriceps ve Hamstring Kas Kuvvetlerinin İzokinetik Sistemle Değerlendirilmesi ve Sakatlık Eğilimlerinin Araştırılması. Master’s Thesis.

[19] Dauty M., Potiron-Josse M., Rochcongar P. (2003). Identification of previous hamstring muscle injury by isokinetic concentric and eccentric torque measurement in elite soccer player. Isokinet. Exerc. Sci..

[20] Ulusoy B. (2014). Hamstring Otogreft İle Ön Çapraz Bağ Rekonstrüksiyonu Sonrası Izokinetik Diz Kuvveti İle Dinamik Denge Arasındaki Ilişkinin Araştırılması. Master’s Thesis.

[21] Ageberg E., Roberts D., Holmstrom E., Friden T. (2005). Balance in singlelimb stance in patients with anterior cruciate ligament injury: Relation to knee laxity, proprioception, muscle strength, and subjective function. Am. J. Sports Med..

[22] Lee H.M., Cheng C.K., Liau J.J. (2009). Correlation between Proprioception, Muscle Strength, Knee Laxity, and Dynamic Standing Balance İn Patients with Chronic Anterior Cruciate Ligament Deficiency. Knee.

[23] McLeod T.C.V., Armstrong T., Miller M., Sauers J.L. (2009). Balance Improvements in Female High School Basketball Players after a 6-Week Neuromuscular-Training Program. J. Sport Rehabil..

[24] Koenig J.P., Puckree T. (2015). Injury Prevalence, Stability and Balance among Female Adolescent Soccer Players: Sport Injury. AJPHERD.

